# Combining use of Phillyrin and autophagy blocker alleviates laryngeal squamous cell carcinoma via AMPK/mTOR/p70S6K signaling

**DOI:** 10.1042/BSR20190459

**Published:** 2019-06-20

**Authors:** Da-hua Wang, Xi He, Qing He

**Affiliations:** 1 Department of Otolaryngology, Xuzhou Municipal Hospital Affiliated with Xuzhou Medical University, Xuzhou 221002, China; 2 Department of Dermatology, Xuzhou Municipal Hospital Affiliated with Xuzhou Medical University, Xuzhou 221002, China; 3 Department of Neurology, Xuzhou Municipal Hospital Affiliated with Xuzhou Medical University, Xuzhou 221002, China

**Keywords:** 3-methyladenine, autophagy, chloroquine, laryngeal squamous cell carcinoma, Phillyrin

## Abstract

Phillyrin (PHN), one of the major active constituents of *Forsythia suspensa* and *F. koreana*, has been reported to produce antioxidant, antibacterial, anti-obesity and anti-inflammatory effects. However, no study has demonstrated the role of PHN in laryngeal squamous cell carcinoma (LSCC). We aimed to investigate the effects of PHN on the proliferation and apoptosis of HEp-2 cells. In the present study, PHN alone showed little effect on HEp-2 cell proliferation and apoptosis. Subsequent tests showed that PHN could largely enhance the level of autophagy on HEp-2 cells. Combining use of PHN and autophagy blockers including 3-methyladenine (3-MA) and chloroquine (CQ) significantly inhibited HEp-2 cell proliferation in a dose- and time-dependent manner and induced apoptosis after 24 h in a dose-dependent manner. Additionally, we found that the possible underlying molecular mechanism of PHN-induced autophagy might be through the AMPK/mTOR/p70S6K signaling pathway. Taken together, our study indicates that combining use of PHN and autophagy blockers may serve as a novel strategy in LSCC treatment.

## Introduction

Laryngeal squamous cell carcinoma (LSCC) is regarded as one of the most common carcinomas of the head and neck [1,2]. So far, surgery, radiotherapy and chemotherapy are applied as the main treatments. However, although the therapeutic effect is moderate in early-stage LSCC, yet it is not as good in advanced stages, despite improvements in the management and treatment of the disease [3,4]. Therefore, it is crucial and urgent for us to gain a better understanding of the molecular mechanisms of LSCC progression and the identification of more effective targets to improve therapeutic efficacy for this disease.

**Figure 1:**
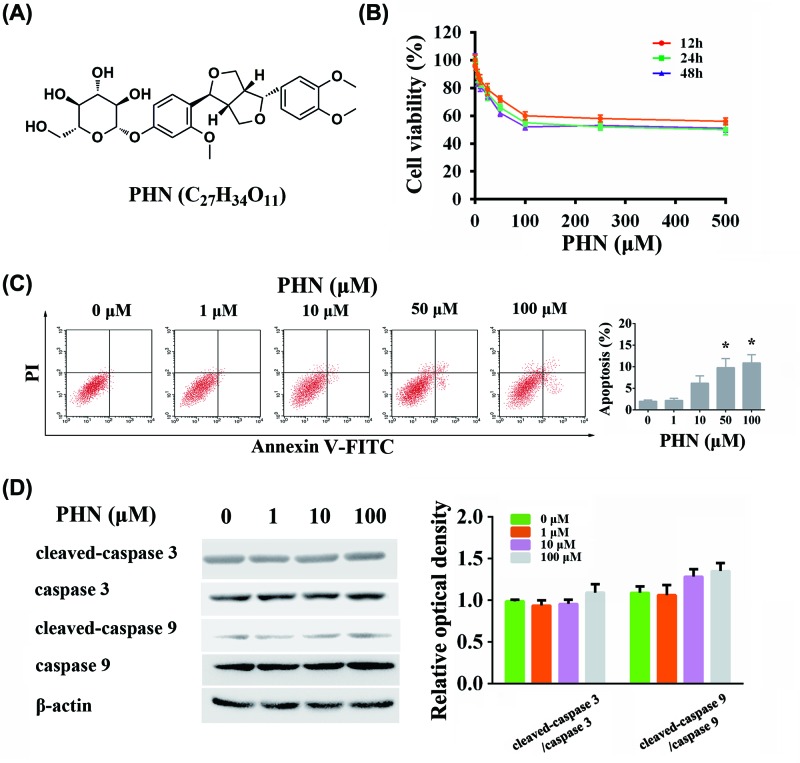
Effects of PHN alone in HEp-2 cells (**A**) Chemical structure of PHN. (**B**) Viability of HEp-2 cells treated with various concentrations of PHN for 12 h (orange), 24 h (green) and 48 h (purple) by CCK8 analysis. (**C**) HEp-2 cells were stained with FITC-Annexin V/PI and analyzed by flow cytometry, with different concentrations of PHN (0, 1, 10, 50 and 100 μM) treatment for 24 h. (**D**) Proteins associated with apoptosis were analyzed by Western blot. The expression level of cleaved-caspase 3, caspase 3, cleaved-caspase 9, caspase 9, β-actin and quantitation of cleaved-caspase 3/caspase 3 and cleaved-caspase 9/caspase 9 ratios. The experiment was repeated three times independently and data are expressed as mean ± standard error, *P<0.05. CCK-8, Cell counting Kit-8; PI, Propidium iodide.

Phillyrin (PHN, shown in Figure 1A), a lignin derivative from *Forsythia suspensa* (Thunb.) Vahl, exerts antioxidant, antibacterial, anti-obesity and anti-inflammatory effects [5–14]. The anti-inflammatory effects of PHN have been increasingly studied recently. So far, several pathways have been reported in the anti-inflammatory process, such as inhibiting cyclo-oxygenase pathway, reducing nitric oxide produced by LPS-stimulated macrophages and ameliorating PAK-induced upper respiratory tract infection [8,9,13]. With the development of epidemiology and advancement of molecular biology techniques, an increasing number of studies have shown close connection between inflammation and tumors [15,16]. Various inflammatory factors have been shown to influence the occurrence and metastasis of tumors by participating and changing the formation of the microenvironment [17,18]. However, so far, no report was available on the anti-tumor effect of PHN.

Autophagy is the process of transporting damaged, denatured or aging proteins and organelles into lysosomes for digestion and degradation [19,20]. Under normal physiological conditions, cellular autophagy promotes the autostable state of cells. However, in the presence of stress, autophagy prevents the accumulation of toxic or carcinogenic damaged proteins and organelles, inhibiting cell carcinogenesis [21,22]. Once the tumor is formed, autophagy provides more nutrients for cancer cells and promotes tumor growth [23]. Moreover, some studies have demonstrated that anti-tumor drugs could trigger potent autophagy thereby affecting the drug effect [24]. Therefore, many studies have combined anti-tumor drugs with autophagy inhibitors to achieve the desired therapeutic effect [25,26]. In this study, we hypothesized that treatment with PHN alone or combined with an autophagy inhibitor could be a potential therapeutic strategy against LSCC.

In the present study, we explored the role of PHN in Hep-2 cells and detected the anti-tumor effect of PHN combined with autophagy inhibitors. Underlying molecular mechanisms were also investigated. We believe that our study provides scientific evidence for the therapeutic application of PHN in human laryngeal epidermoid carcinoma.

## Materials and methods

### Reagents

PHN (HPLC > 98%, Kay LiDe Biomedical Technology, Shanghai, China), compound C (CC) (Sigma–Aldrich Chemical, St. Louis, U.S.A.), chloroquine (CQ) (Sigma–Aldrich Chemical, St. Louis, U.S.A.) and 3-methyladenine (3-MA) (Sigma–Aldrich, St. Louis, U.S.A.) were dissolved in dimethyl sulfoxide (DMSO) and kept at −20°C. Cleaved caspase 3 (#9661), caspase 3 (#9662), cleaved caspase 9 (#9505), caspase 9 (#9502), BECLIN-1 (#3495), LC3 (#12741), P62 (#8025), P-AMPK (#2535), AMPK (#2532), P-mTOR (#2971), mTOR (#2972), P-p70S6K (#9208), p70S6K (#9202) and β-actin (#3700) were purchased from Cell Signaling Technology (MA, U.S.A.).

### Cell lines and cell culture

HEp-2 cells delivered from a human laryngeal epidermoid carcinoma of the larynx (CCL-23, ATCC, U.S.A.) were cultured in Dulbecco’s modified Eagle’s medium (DMEM) supplemented with 10% fetal bovine serum (FBS; Gibco, U.S.A.) and 1% streptomycin–penicillin and maintained at 37°C in 5% carbon dioxide.

### Cell proliferation assay

HEp-2 cells were seeded into a 96-well plate at a density of 1.0 × 10^5^ cells/well overnight and then subjected to various indicated treatments. Cell viability was measured by a CCK-8 (Dojindo Molecular Technologies, Japan).

### Apoptosis assay

An Annexin V-FITC/PI Apoptosis Detection Kit (BD Bioscience, U.S.A.) was employed to measure apoptosis. HEp-2 cells were collected and washed with PBS and then resuspended in 500 μl binding buffer at a concentration of 1 × 10^6^ cells/ml. Subsequently, the cells were incubated with Annexin V-FITC and PI for 15 min. Data were analyzed by an FACSCalibur flow cytometer.

### Western blot analysis

Following cell lysis, proteins were resolved by SDS/PAGE, transferred to PVDF membranes and incubated with primary antibodies specific to the protein of interest. Antigen–antibody complexes were visualized using secondary horseradish peroxidase–conjugated antibodies. The immunoreactive bands were detected using an Enhanced Chemiluminescence Detection Kit (Sigma–Aldrich).

### Statistics

Statistical analyses were performed using GraphPad Prism 6 software (GraphPad Prism, San Diego, CA, U.S.A.). All results are depicted as the mean ± standard error of the mean (SEM) from at least three independent experiments. Differences were considered statistically significant when *P*<0.05 using two-way ANOVA.

## Results

### Application of PHN alone does not effectively inhibit HEp-2 cells

CCK-8 assay was used to determine the effect of PHN on cell viability. PHN inhibited HEp-2 cell viability, albeit weakly. Even the concentration reaching 500 μM, only approximately 50% of cells were inhibited (Figure 1B). In addition, with the increase in PHN in concentration, the cell viability of HEp-2 cells decreased. However, after reaching 100 μM, the effect of PHN did not change (Figure 1B). Based on those results, in the subsequent experiments, we set the maximum PHN concentration to 100 μM. Moreover, we found that after PHN was incubated with HEp-2 cells at three time points (12, 24 and 48 h), PHN showed time-dependent effects. However, there was little difference in the intensity of action between 24 and 48 h (Figure 1B). Therefore, in the latter experiments, we mainly used 24 h as the time point for testing.

### Caspase 3/caspase 9-dependent apoptosis was slightly activated in PHN-treated HEp-2 cells

In addition to cell viability assays, we also examined the effects of PHN-induced caspase 3/caspase 9-dependent apoptosis. The results were similar to those obtained from cell viability tests (Figure 1C). At 24 h, PHN only slightly induced apoptosis with increasing concentrations. We found that approximately 10% of cells underwent apoptosis at 100 μM, which was similar in the effect of PHN on apoptosis at the concentration of 50 μM (Figure 1C). In addition, we also examined the level of caspase 3/caspase 9-dependent apoptosis. We found that changes in cleaved-caspase 3/caspase 3 and cleaved-caspase 9/caspase 9 did not significantly differ along with the increase in concentration (Figure 1D). These results indicated that PHN could induce apoptosis, albeit to a weak extent.

### Autophagy was induced by PHN in HEp-2 cells

**Figure 2:**
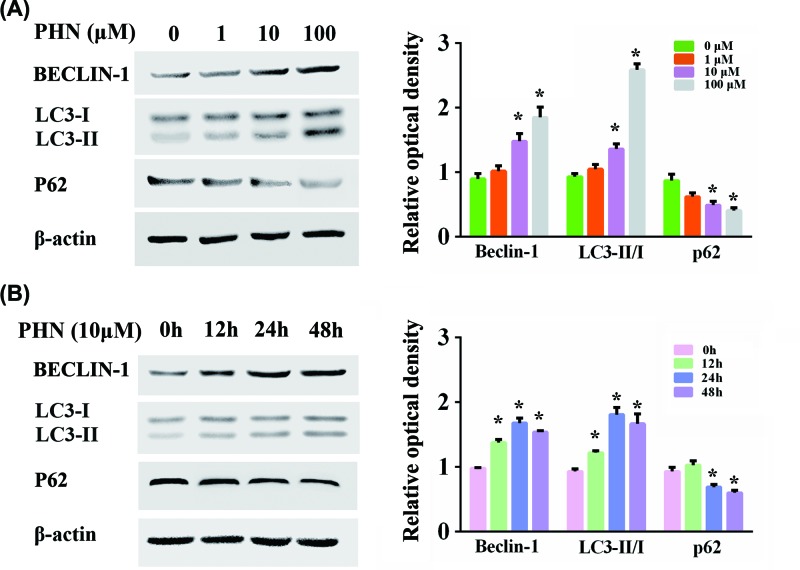
PHN triggered autophagy in HEp-2 cells (**A**) Proteins associated with autophagy were analyzed by Western blot with different concentrations of PHN (0, 1, 10 and 100 μM) treatment for 24 h. The expression level of BECLIN-1, LC3II/I and P62 and quantitation of the relative protein expression. (**B**) Proteins associated with autophagy were analyzed by Western blot with 10 μM PHN treatment for different times (0, 12, 24 and 48 h). The expression levels of BECLIN-1, LC3II/I ratio and P62 and quantitation of the relative protein expression. The experiment was repeated three times independently and data are expressed as mean ± standard error, *P<0.05.

Autophagy has been reported to be closely connected to inflammation. Since PHN was previously shown to produce an anti-inflammatory effect, we detected the association between PHN and autophagy. We examined the expressions of autophagy-related proteins including BECLIN-1, LC3II/I and P62 (Figure 2). The levels of BECLIN-1 and LC3 II/I ratio were up-regulated with the increase in PHN concentration at 24 h, while that of P62 was down-regulated, indicating the increase in the levels of autophagy in a dose-dependent manner of PHN (Figure 2A). In particular, at a concentration of 10 μM, PHN significantly enhanced autophagy. We also tested the effects of PHN at 12, 24 and 48 h at the concentration of 10 μM (Figure 2B). We found that the level of autophagy was highest with the stimulation for 24 h.

### 3-MA enhanced PHN-induced cytotoxicity in HEp-2 cells

**Figure 3:**
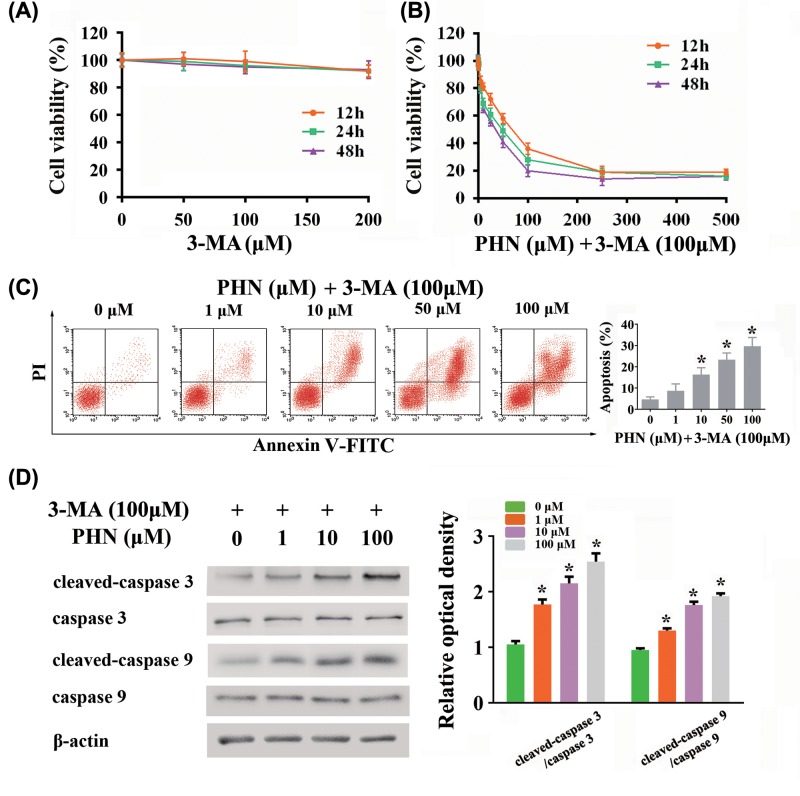
Effects of combining use of PHN and 3-MA in HEp-2 cells (**A**) Viability of HEp-2 cells treated with various concentrations of 3-MA for 12 h (orange), 24 h (green) and 48 h (purple) by CCK8 analysis. (**B**) Viability of HEp-2 cells treated with various concentrations of PHN and 100 μM 3-MA for 12 h (orange), 24 h (green) and 48 h (purple) by CCK8 analysis. (**C**) HEp-2 cells were stained with FITC-Annexin V/PI and analyzed by flow cytometry, with different concentrations of PHN (0, 1, 10, 50 and 100 μM) and 100 μM 3-MA treatment for 24 h. (**D**) Proteins associated with apoptosis were analyzed by Western blot. The expression level of cleaved-caspase 3, caspase 3, cleaved-caspase 9, caspase 9, β-actin and quantitation of cleaved-caspase 3/caspase 3 and cleaved-caspase 9/caspase 9 ratios. The experiment was repeated three times independently and data are expressed as mean ± standard error, *P<0.05.

As shown above, PHN could induce autophagy on HEp-2 cells. We then investigated the effects of combining use of PHN and an autophagy blocker on cytotoxity on HEp-2 cells with the use of the autophagy inhibitor, 3-MA. In the first set of experiments, 3-MA was used for the blockade of autophagy. We first ruled out the effect of 3-MA on the viability of HEp-2 cells (Figure 3A). We found that at different incubation times (12, 24 and 48 h) respectively, different concentrations of 3-MA (50, 100, 200 μM) did not affect the viability of HEp-2 cells. Therefore, we chose 100 μM as the concentration of 3-MA in use. We found that combining use of PHN and 3-MA significantly inhibited the viability of HEp-2 cells in a dose-dependent manner (IC_50_ = 57.66 ± 19.98 μM for 12 h, IC_50_ = 26.91 ± 5.59 μM for 24 h, IC_50_ = 23.32 ± 4.85 μM for 48 h) (Figure 3B). In addition, along with the prolonging of PHN incubation time, the effect on cell proliferation inhibition was gradually strengthened in a time-dependent manner. We then tested the effect of combining use of PHN and 3-MA at 24 h on apoptosis. Combining use of PHN and 3-MA at 24 h significantly enhanced the level of apoptosis in HEp-2 cells in a dose-dependent manner (Figure 3C). Furthermore, we also examined caspase 3/caspase 9-dependent apoptosis-related proteins. We found that cleaved-caspase 3/caspase 3 and cleaved-caspase 9/caspase 9 ratios were up-regulated in a dose-dependent manner after 24 h stimulation of PHN and 3-MA (Figure 3D).

### CQ enhanced PHN-induced cytotoxicity in HEp-2 cells

**Figure 4:**
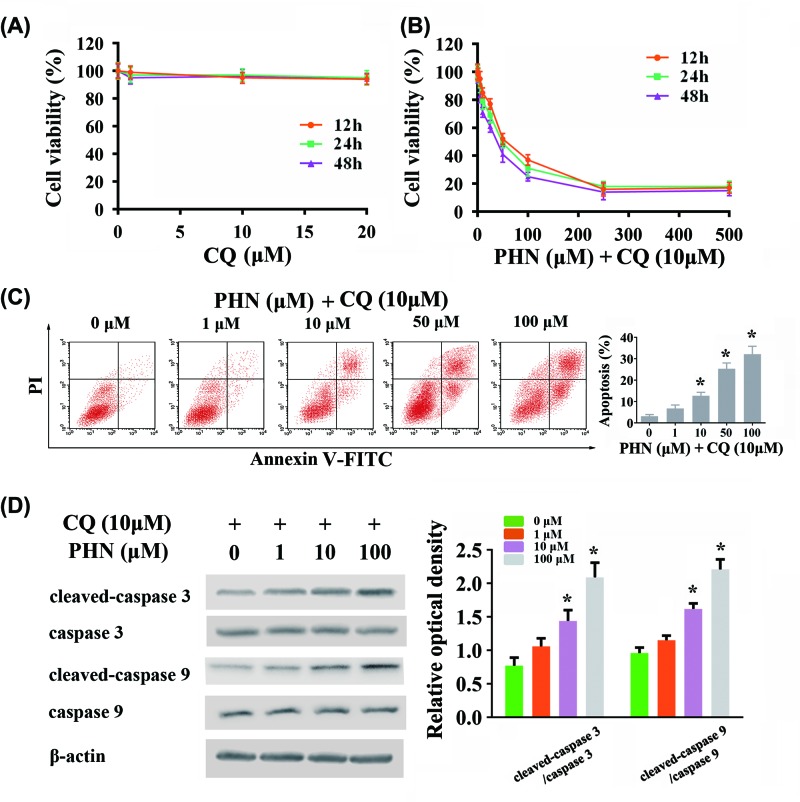
Effects of combining use of PHN and CQ in HEp-2 cells (**A**) Viability of HEp-2 cells treated with various concentrations of CQ for 12 h (orange), 24 h (green) and 48 h (purple) by CCK8 analysis. (**B**) Viability of HEp-2 cells treated with various concentrations of PHN and 10 μM CQ for 12 h (orange), 24 h (green) and 48 h (purple) by CCK8 analysis. (**C**) HEp-2 cells were stained with FITC-Annexin V/PI and analyzed by flow cytometry, with different concentrations of PHN (0, 1, 10, 50 and 100 μM) and 10 μM CQ treatment for 24 h. (**D**) Proteins associated with apoptosis were analyzed by Western blot. The expression level of cleaved-caspase 3, caspase 3, cleaved-caspase 9, caspase 9, β-actin and quantitation of cleaved-caspase 3/caspase 3 and cleaved-caspase 9/caspase 9 ratios. The experiment was repeated three times independently and data are expressed as mean ± standard error, *P<0.05.

For further investigation, we applied another autophagy inhibitor, CQ. We first ruled out the effect of CQ on the viability of HEp-2 cells (Figure 4A). At different incubation times (12, 24 and 48 h) respectively, different concentrations of CQ (1, 10, 20 μM) did not affect the viability of HEp-2 cells. Accordingly, we chose 10 μM for the concentration of CQ in use. We found that combining use of PHN and CQ significantly inhibited the viability of HEp-2 cells in a time- and dose-dependent manner (IC_50_ = 49.16 ± 6.52 μM for 12 h, IC_50_ = 38.29 ± 5.52 μM for 24 h, IC_50_ = 28.90 ± 5.56 μM for 48 h) (Figure 4B). We then tested the effect of combining use of PHN and CQ at 24 h on apoptosis. Combining use of PHN and CQ at 24 h significantly enhanced the level of apoptosis in HEp-2 cells in a dose-dependent manner (Figure 4C). Furthermore, we also examined caspase 3/caspase 9-dependent apoptosis-related proteins. We found that cleaved-caspase 3/caspase 3 and cleaved-caspase 9/caspase 9 ratios were up-regulated in a dose-dependent manner after 24-h stimulation of PHN and CQ (Figure 4D).

### AMPK/mTOR/p70S6K signaling pathway was involved in PHN-mediated autophagy induction in HEp-2 cells

**Figure 5:**
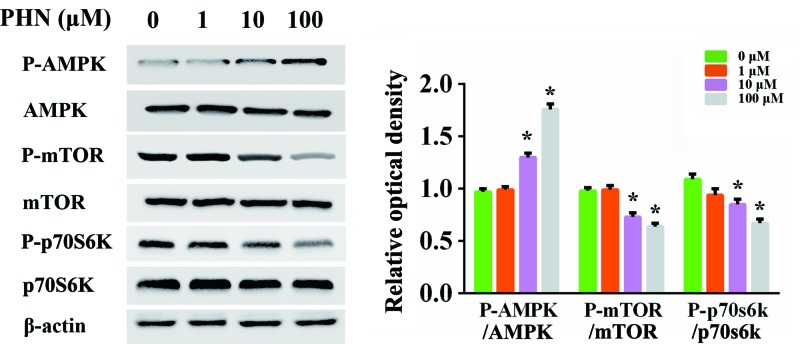
AMPK/mTOR/p70S6K signaling pathway was implicated in PHN-induced autophagy HEp-2 cells were treated with various concentrations of PHN (0, 1, 10 and 100 μM) for 24 h, and the expression of proteins associated with autophagy signal pathways was analyzed by Western blot. The expression levels of P-AMPK, AMPK, P-mTOR, mTOR, P-p70S6K, p70S6K, β-actin and quantitation of P-AMPK/AMPK, P-mTOR/mTOR and P-p70S6K/p70S6K ratios. The experiment was repeated three times independently and data are expressed as mean ± standard error, *P<0.05.

We then detected the molecular mechanism underlying. We investigated whether AMPK/mTOR/p70S6K signaling, the classic autophagy-related pathway, was involved in this process. We found that the level of P-AMPK/AMPK ratio was increased in a dose-dependent manner after incubation with PHN for 24 h, while the levels of P-mTOR/mTOR and P-p70S6K/p70S6K ratios were decreased (Figure 5). These results suggested that AMPK/mTOR/p70S6K pathway was involved in PHN-mediated induction of autophagy.

### Blocking AMPK increased anti-tumor activity of PHN

**Figure 6:**
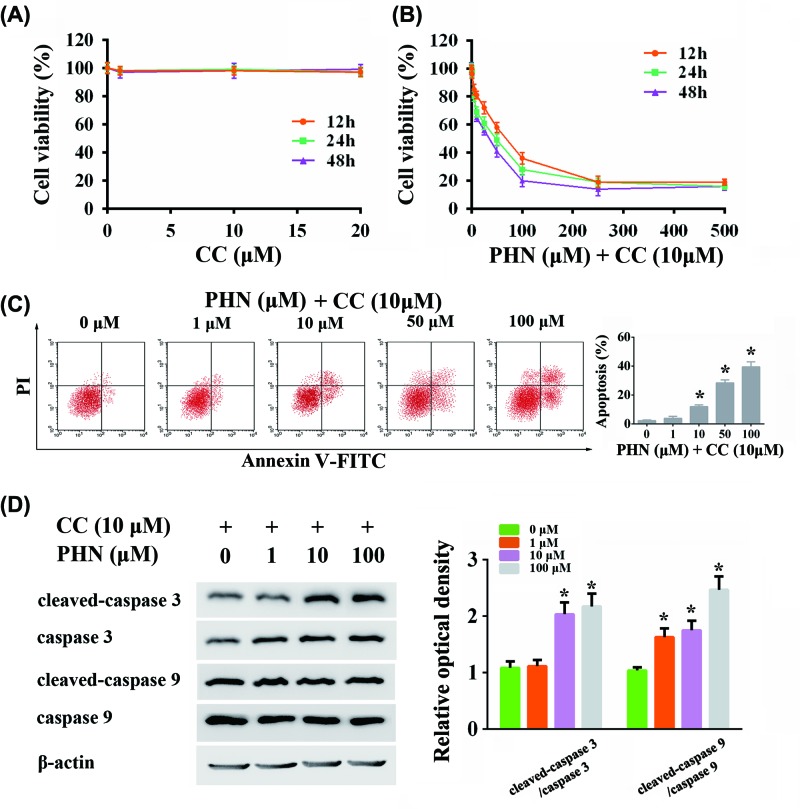
Effects of combining use of PHN and CC in HEp-2 cells (**A**) Viability of HEp-2 cells treated with various concentrations of CC for 12 h (orange), 24 h (green) and 48 h (purple) by CCK8 analysis. (**B**) Viability of HEp-2 cells treated with various concentrations of PHN and 10 μM CC for 12 h (orange), 24 h (green) and 48 h (purple) by CCK8 analysis. (**C**) HEp-2 cells were stained with FITC-Annexin V/PI and analyzed by flow cytometry, with different concentrations of PHN (0, 1, 10, 50 and 100 μM) and 10 μM CC treatment for 24 h. (**D**) Proteins associated with apoptosis were analyzed by Western blot. The expression level of cleaved-caspase 3, caspase 3, cleaved-caspase 9, caspase 9 and quantitation of cleaved-caspase 3/caspase 3 and cleaved-caspase 9/caspase 9 ratios. The experiment was repeated three times independently and data are expressed as mean ± standard error, *P<0.05.

To further verify whether PHN-induced autophagy was via AMPK/mTOR/p70S6K pathway, we used AMPK inhibitor, CC. We first found that there was no cytotoxic effect when CC was incubated with HEp-2 cells alone at the concentrations of 0–20 μm (Figure 6A). Accordingly, the concentration of 10 μm was used in the substantial experiments. Along with blocking AMPK with CC, we found that the administration of PHN significantly inhibited the viability of HEp-2 cells in a time- and dose-dependent manner (IC_50_ = 53.17 ± 15.53 μM for 12 h, IC_50_ = 29.94 ± 8.72 μM for 24 h, IC_50_ = 20.37 ± 4.97 μM for 48 h) (Figure 6B). We then tested the effect of combining use of PHN and CC at 24 h on apoptosis. Combining use of PHN and CC at 24 h significantly enhanced the level of apoptosis in HEp-2 cells in a dose-dependent manner (Figure 6C). Furthermore, we also examined caspase 3/caspase 9-dependent apoptosis-related proteins. We found that cleaved-caspase 3/caspase 3 and cleaved-caspase 9/caspase 9 ratios were up-regulated in a dose-dependent manner after 24-h stimulation of PHN and CC (Figure 6D).

## Discussion

Currently, surgery is regarded as the primary treatment for LSCC. In addition, nonsurgical options like radiation and chemotherapy have emerged as viable options [27]. However, tumor cells resistance to chemotherapeutic drugs tend to result in tumor recurrence [28]. Therefore, it is necessary to find more effective anti-tumor drugs with other mechanisms. PHN is one of the major active constituents of *F. suspensa* and *F. koreana*. In recent years, an increasing number of studies have shown that PHN has anti-inflammatory effects [8–14]. PHN was initially reported that it could exert a preferential effect on the cyclo-oxygenase pathway, inhibiting release of the cyclo-oxygenase metabolites prostaglandin E2 and to a lesser extent reducing thromboxane B2 levels [9]. Moreover, PHN could decrease neutrophil infiltration, reduce tissue necrosis and increase survival rates [10]. It was also reported that the intestinal metabolite of PHN exhibited anti-inflammatory activity through modulating multiple cellular behaviors, leading to the suppression of adaptive immune response [14]. At present, PHN has been widely used in clinical traditional Chinese medicine treatment. Its main functions include clearing away heat, detoxifying, reducing swelling and dispersing. However, so far, the anti-tumor effects of PHN have not been reported. Here, we reported that PHN combined with autophagy blockers could produce anti-tumor effects at least partially through inhibition of autophagy via AMPK/mTOR/p70S6K signaling pathway, suggesting that combining use of PHN and an autophagy blocker might serve as a novel strategy against LSCC.

Autophagy is an evolutionarily conserved catabolic process that directs cytoplasmic proteins, organelles and microbes to lysosomes for degradation. An increasing number of studies have reported that autophagy has a protective effect on the occurrence and development of tumors [29,30]. Tumor cells are usually in a state of hypoxia and nutrient factor deficiency which could induce autophagy. Meanwhile, autophagy can provide tumor cells with the nutrients to promote their survival [31–33]. Therefore, here in the present study, we used autophagy inhibitors, 3-MA and CQ, to block PHN-induced autophagy, which could reduce the nutritional supply to tumor cells. We found that the ability to inhibit cell viability and to induce apoptosis were significantly improved. Our results also suggested that the reason for the poor activity of some anti-tumor drugs in the current stage may be due to autophagy caused by tumor cells. For these drugs, we could combine autophagy inhibitors to enhance their activity. Base on these findings, our research provides a new idea for the future application of anti-tumor drugs.

However, it should be noted that there are some potential problems using 3-MA as the autophagy blocker. Since high concentration of 3-MA not only inhibits class I phosphoinositide 3-kinase, but also blocks class III phosphatidylinositol 3-kinase, the survival signaling pathways [34]. Therefore, using 3-MA might have a slight increase in autophagy [35]. To be more clear, we additionally used another autophagy inhibitor CQ, a lysosomal degradation blocker, which could inhibit the fusion of autophagosomes and lysosomes [36,37]. We believe that 3-MA and CQ are more illustrative for our conclusions.

We finally investigated the specific molecular mechanism of the anti-tumor effect of PHN combined with autophagy inhibitors and focused on AMPK/mTOR/p70S6K signaling pathway. It has been reported that AMPK/mTOR/p70S6K signaling pathway plays a role in tumor development [38,39]. Consistent with those findings, here we demonstrated that the induction of autophagy by PHN were at least partly through the AMPK/mTOR/p70S6K signaling pathway. Since the mechanisms of autophagy process is very complicated, further studies are demanded on this issue.

Collectively, our current study demonstrated for the first time that PHN produced effects on inducing autophagy and anti-tumor activity on HEp-2 cells, in which autophagy induction could offset some of its anti-tumor activity. Combing use of PHN and autophagy blockers exerted enhanced anti-tumor activity, which at least partially through the AMPK/mTOR/p70S6K signal pathway. Our research provides scientific evidence for the therapeutic application of PHN in human laryngeal epidermoid carcinoma.

## Abbreviations

CC: compound C
CQ: chloroquine
LSCC: laryngeal squamous cell carcinoma
PHN: Phillyrin
3-MA: 3-methyladenine

## References

[1] Fanjul-Fernandez M., Quesada V., Cabanillas R., Cadinanos J. (2013) Cell-cell adhesion genes CTNNA2 and CTNNA3 are tumour suppressors frequently mutated in laryngeal carcinomas, Nat Commun. 4, 2531 10.1038/ncomms3531 24100690

[2] De Vito R., Lee Y.C.A., Parpinel M., 2019 Shared and study-specific dietary patterns and head and neck cancer risk in an international consortium, Epidemiology 30, 93–102, 10.1097/EDE.0000000000000902 30063539 PMC6269206

[3] Wu Y.H. and Tang P.Z. (2006) Current opinions in therapeutics of advanced laryngeal carcinoma. Zhong. Yi Xue Ke Xue Yuan Xue Bao 28, 431–434 16900651

[4] Gourin C.G., Conger B.T., Sheils W.C. (2009) The effect of treatment on survival in patients with advanced laryngeal carcinoma. Laryngoscope 119, 1312–1317 10.1002/lary.20477 19444887

[5] Qu H., Zhang Y., Wang Y. (2008) Antioxidant and antibacterial activity of two compounds (forsythiaside and forsythin) isolated from *Forsythia suspensa*. J. Pharm. Pharmacol. 60, 261–266 10.1211/jpp.60.2.0016 18237475

[6] Do M.T., Kim H.G., Choi J.H. (2013) Phillyrin attenuates high glucose-induced lipid accumulation in human HepG2 hepatocytes through the activation of LKB1/AMP-activated protein kinase-dependent signalling. Food Chem. 136, 415–425 10.1016/j.foodchem.2012.09.012 23122079

[7] Zhao Y., Li F., Yang J. (2005) Effect of phillyrin on the anti-obesity in nutritive obesity mice. Zhong Yao Cai 28, 123–124 15981888

[8] Lee D.G., Lee S.M., Bang M.H. (2011) Lignans from the flowers of *Osmanthus fragrans* var. aurantiacus and their inhibition effect on NO production. Arch. Pharm. Res. 34, 2029–2035 10.1007/s12272-011-1204-y 22210027

[9] Diaz Lanza A.M., Abad Martinez M.J., Fernandez Matellano L. (2001) Lignan and phenylpropanoid glycosides from *Phillyrea latifolia* and their *in vitro* anti-inflammatory activity. Planta Med. 67, 219–223 10.1055/s-2001-12004 11345691

[10] Yang L., Zhou X., Huang W. (2017) Protective effect of phillyrin on lethal LPS-induced neutrophil inflammation in zebrafish. Cell. Physiol. Biochem. 43, 2074–2087 10.1159/000484192 29059681

[11] Kong P., Zhang L., Guo Y. (2014) Phillyrin, a natural lignan, attenuates tumor necrosis factor alpha-mediated insulin resistance and lipolytic acceleration in 3T3-L1 adipocytes. Planta Med. 80, 880–886 10.1055/s-0034-1368614 24995500

[12] Zhong W.T., Wu Y.C., Xie X.X. (2013) Phillyrin attenuates LPS-induced pulmonary inflammation via suppression of MAPK and NF-kappaB activation in acute lung injury mice. Fitoterapia 90, 132–139 10.1016/j.fitote.2013.06.003 23751215

[13] Li Y., Chang N., Han Y. (2017) Anti-inflammatory effects of Shufengjiedu capsule for upper respiratory infection via the ERK pathway. Biomed. Pharmacother. 94, 758–766 10.1016/j.biopha.2017.07.118 28802227

[14] Du B., Zhang L., Sun Y. (2019) Phillygenin exhibits anti-inflammatory activity through modulating multiple cellular behaviors of mouse lymphocytes. Immunopharmacol. Immunotoxicol. 41, 76–85 10.1080/08923973.2018 30721636

[15] Albrengues J. and Shields M.A. (2018) Neutrophil extracellular traps produced during inflammation awaken dormant cancer cells in mice, Science 361, 6409 10.1126/science.aao4227 PMC6777850 30262472

[16] Dou Z., Ghosh K., Vizioli M.G. (2017) Cytoplasmic chromatin triggers inflammation in senescence and cancer. Nature 550, 402–406 10.1038/nature24050 28976970 PMC5850938

[17] Dmitrieva-Posocco O., Dzutsev A., Posocco D.F. (2019) Cell-type-specific responses to interleukin-1 control microbial invasion and tumor-elicited inflammation in colorectal cancer. Immunity 50, 166–180.e167 10.1016/j.immuni.2018.11.015 30650375 PMC6490968

[18] Sakthianandeswaren A., Parsons M.J., Mouradov D. (2018) MACROD2 haploinsufficiency impairs catalytic activity of PARP1 and promotes chromosome instability and growth of intestinal tumors, Cancer Discov. 8, 988–1005 10.1158/2159-8290 29880585

[19] Levine B. and Kroemer G. (2019) Biological functions of autophagy genes: a disease perspective. Cell 176, 11–42 10.1016/j.cell.2018.09.048 30633901 PMC6347410

[20] Clarke A.J. and Simon A.K. (2019) Autophagy in the renewal, differentiation and homeostasis of immune cells, Nat Rev Immunol. 19, 170–183 10.1038/s41577-018-0095-2 30531943

[21] Rybstein M.D., Bravo-San Pedro J.M., Kroemer G. (2018) The autophagic network and cancer. Nat. Cell Biol. 20, 243–251 10.1038/s41556-018-0042-2 29476153

[22] Galluzzi L., Bravo-San Pedro J.M., Demaria S. (2017) Activating autophagy to potentiate immunogenic chemotherapy and radiation therapy. Nat. Rev. Clin. Oncol. 14, 247–258 10.1038/nrclinonc.2016.183 27845767

[23] Perera R.M. and Bardeesy N. (2015) Pancreatic cancer metabolism: breaking it down to build it back up. Cancer Discov. 5, 1247–1261 10.1158/2159-8290.CD-15-0671 26534901 PMC4687899

[24] Gozuacik D. and Kimchi A. (2004) Autophagy as a cell death and tumor suppressor mechanism. Oncogene 23, 2891–2906 10.1038/sj.onc.1207521 15077152

[25] Ji Y., Li L., Tao Q. (2017) Deprivation of asparagine triggers cytoprotective autophagy in laryngeal squamous cell carcinoma. Appl. Microbiol. Biotechnol. 101, 4951–4961 10.1007/s00253-017-8221-9 28352997

[26] Liu H., Gu L.B., Tu Y. (2016) Emodin ameliorates cisplatin-induced apoptosis of rat renal tubular cells in vitro by activating autophagy. Acta Pharmacol. Sin. 37, 235–245 10.1038/aps.2015.114 26775661 PMC4753365

[27] Steuer C.E., El-Deiry M., Parks J.R. (2017) An update on larynx cancer. CA Cancer J. Clin. 67, 31–50 10.3322/caac.21386 27898173

[28] Leeman J.E., Li J.G., Pei X. (2017) Patterns of treatment failure and postrecurrence outcomes among patients with locally advanced head and neck squamous cell carcinoma after chemoradiotherapy using modern radiation techniques. JAMA Oncol. 3, 1487–1494 10.1001/jamaoncol.2017.0973 28542679 PMC5710194

[29] Gomes L.C., Odedra D., Dikic I. (2016) Autophagy and modular restructuring of metabolism control germline tumor differentiation and proliferation in C. elegans. Autophagy 12, 529–546 10.1080/15548627.2015.1136771 26759963 PMC4835962

[30] White E. (2012) Deconvoluting the context-dependent role for autophagy in cancer. Nat. Rev. Cancer 12, 401–410 10.1038/nrc3262 22534666 PMC3664381

[31] Mazure N.M. and Pouyssegur J. (2010) Hypoxia-induced autophagy: cell death or cell survival? Curr. Opin. Cell Biol. 22, 177–180 10.1016/j.ceb.2009.11.015 20022734

[32] Fang Y., Tan J. and Zhang Q. (2015) Signaling pathways and mechanisms of hypoxia-induced autophagy in the animal cells. Cell Biol. Int. 39, 891–898 10.1002/cbin.10463 25808799

[33] Paredes-Juarez G.A., Sahasrabudhe N.M., Tjoelker R.S. (2015) DAMP production by human islets under low oxygen and nutrients in the presence or absence of an immunoisolating-capsule and necrostatin-1. Sci. Rep. 5, 14623 10.1038/srep14623 26419792 PMC4588515

[34] Mizushima N., Yoshimori T. and Levine B. (2010) Methods in mammalian autophagy research. Cell 140, 313–326 10.1016/j.cell.2010.01.028 20144757 PMC2852113

[35] Wu Y.T., Tan H.L., Shui G. (2010) Dual role of 3-methyladenine in modulation of autophagy via different temporal patterns of inhibition on class I and III phosphoinositide 3-kinase. J. Biol. Chem. 285, 10850–10861 10.1074/jbc.M109.080796 20123989 PMC2856291

[36] Radhika A. and Sudhakaran P.R. (2013) Upregulation of macrophage-specific functions by oxidized LDL: lysosomal degradation-dependent and -independent pathways. Mol. Cell. Biochem. 372, 181–190 10.1007/s11010-012-1459-8 23054190

[37] Ju J.S., Varadhachary A.S., Miller S.E. (2010) Quantitation of “autophagic flux” in mature skeletal muscle. Autophagy 6, 929–935 10.4161/auto.6.7.12785 20657169 PMC3039739

[38] Song L., Wang Z., Wang Y. (2017) Natural cyclopeptide RA-XII, a new autophagy inhibitor, suppresses protective autophagy for enhancing apoptosis through AMPK/mTOR/P70S6K pathways in HepG2 cells. Molecules 22, 11 10.3390/molecules22111934 PMC6150396 29137114

[39] Liu Z., Ren L., Liu C. (2015) Phenformin induces cell cycle change, apoptosis, and mesenchymal-epithelial transition and regulates the AMPK/mTOR/p70s6k and MAPK/ERK pathways in breast cancer cells. PLoS ONE 10, e0131207 10.1371/journal.pone.0131207 26114294 PMC4482683

